# Supplementary material for: SESN3 Inhibited SMAD3 to Relieve Its Suppression for MiR-124, Thus Regulating Pre-Adipocyte Adipogenesis
Source: Genes (Basel). 2021 Nov 23;12(12):1852. doi: 10.3390/genes12121852 (PMC8701261; doi:10.3390/genes12121852)
Supplement: Supplementary file 1 [file genes-12-01852-s001.zip › genes-1445016-supplementary-done.pdf]

genes

Supplementary Table S1. The sequence used in the whole paper.

Name	Sequence (5'- 3')	Length (bp)	TM/°C
C/EBPα qPCR	F:CCTTCAACGACGAGTTCCTGG<br>R:CTGCTTCGACTCGTCCTCCTC	296	59.5
C/EBPβ qPCR	F:TAAGCACAGCGACGAGTACAAGA<br>R:TTGAACAAGTTCCGCAGGGT	192	60
FABP4 qPCR	F:GACAGGAAAGTCAAGAGCACCA<br>R:TCGGGACAATACATCCAACAGAG	229	60
PPARγ qPCR	F:CAGAGTATGCCAAGAACATCCCT<br>R:GCAAACTCGAACTTGGGCTC	223	58.5
FoxO1 qPCR	F:CTGTCCTACGCCGACCTCA<br>R:CCCACTCTTGCCTCCCTCT	255	60.5
Sesn3 qPCR	F:TGGTCCTGCTGGCACATTAT<br>R: GACGCCTCTTCATCTTCCCT<br>R: TCAGTGCATTGCCCTTGTCA	265	59.5
GR qPCR	F: GAAGGGGGCGGCTGTTTA<br>R: ATCAGTGGGTATCAGCTCTGC	155	60
Smad1 qPCR	F: GCCAACCCGGTGCTGA<br>R: CCTCGTGGACGGAGAAACAA	169	59.5
Smad2 qPCR	F: ATGTGTTACCATACCAAGGTCCC<br>R: ATGCCGTGGCACTAATACTGG	241	60.5
Smad3 qPCR	F: GGAGGAGAAGTGGTGCGAGA<br>R: CACAGGCGGCAGTAGATGACA	198	59.5
Smad4 qPCR	F: CGCTGGAGCCCAGGTTATC<br>R: ACAATGCTCAGACAGGCATCA	201	60
Smad5 qPCR	F: GAGGCTTCCTCAGCCGC<br>R: TTTCATTGGTTCAAGTCGGGC	248	60.5
Smad6 qPCR	F: CTCTGCGGGCCAGAATCAC<br>R: GTGGCCTCCGTTTCAGTGTA	113	60
Smad7 qPCR	F: GTGCAAAGTGTTCAGGTGGC<br>R: CACAGCATCTGGACAGTCAGT	208	60
Smad9 qPCR	F: ACACATAGGAAAGGGCGTGC<br>R: TTCAAAGCCATGGTGGACGG	220	60
GAPDH qPCR	F: GTTCCAGTATGATTCCACCCAC<br>R: TTCACGCCCATCACAAACAT	270	60
ChIP Smad3-124 Primer 1	F: GCCTTATTTGTTTGGCTGGAG<br>R: GAGGACCAAAGCGTTTTCAATC	198	59.5
ChIP Smad3-124 Primer 2	F: CTGAGCAGCGATTGAAAACG<br>R: CCCCTTCTTGGCTTAGTACCCT	178	61.5
ChIP Smad3-124 Primer 3	F: GACCATCTGCGGACGCTTT<br>R: GAGATAAGGTGACGAGAGGACGA	248	61
	F: TCTCGTCCTCTCGTCACCTTATC	176	60.5

ChIP Smad3-124 Primer 4	R: GGCTGTGGCTGCAGAAACC		
ChIP FoxO1-Sesn3 Primer	F:TCAACACATTTACCCAACCACAT<br>R:CCAATCTCCTCATTTTTTCGACA	183	62
miR-17 qPCR	F: CGGGCCAAAGTGCTTACAGT<br>R:CGCAGGGTCCGAGGTATTC	88	60
miR-124 qPCR	F:GTAAGGCACGCGGTGAATG<br>R: CGCAGGGTCCGAGGTATTC	84	60
si-FoxO1	F:GCAUGUUCAUUGAGCGCUUTT<br>R:AAGCGCUCAAUGAACAUGCTT		
si-Sesn3	F:GACAGUUACUGGCGUCAAUUTT<br>R:AAUUGACGCCAGUAACUGUCTT		
miR-17 RT	GTCGTATCCAGTGCAGGGTCCGAGGTATTCGCACTG<br>GATACGACTCTACC	/	/
miR-124 RT	GTCGTATCCAGTGCAGGGTCCGAGGTATTCGCACTG<br>GATACGACTGGCAT	/	/
miR-124 mimics	F:UAAGGCACGCGGUGAAUGCCA<br>R:GCAUUCACCGCGUGCCUUAUU	/	/
miR-124 inhibitor	UGGCAUUCACCGCGUGCCUUA	/	/
